# Antibiotic prescribing for respiratory tract infection in patients with suspected and proven COVID-19: results from an antibiotic point prevalence survey in Scottish hospitals

**DOI:** 10.1093/jacamr/dlab078

**Published:** 2021-06-18

**Authors:** R Andrew Seaton, Lesley Cooper, Cheryl L Gibbons, William Malcolm, Brian Choo-Kang, David Griffith, Stephanie Dundas, Suzanne Brittain, Kayleigh Hamilton, Danielle Jeffreys, Rachel McKinney, Debbie Guthrie, Jacqueline Sneddon

**Affiliations:** 1 Infectious Diseases Unit, Queen Elizabeth University Hospital, 1345 Govan Road, Glasgow, G51 4TF, UK; 2 Scottish Antimicrobial Prescribing Group, Healthcare Improvement Scotland, Delta House, 48 West Nile Street, Glasgow, G1 2NP, UK; 3 Public Health Scotland, NHS National Services Scotland, Meridian Court, 5 Cadogan Street, Glasgow, G2 6QE, UK; 4 ARHAI Scotland, NHS National Services Scotland, Meridian Court, 5 Cadogan Street, Glasgow, G2 6QE, UK; 5 Respiratory Medicine Unit, Glasgow Royal Infirmary, 84 Castle Street, Glasgow, G4 0SF, UK; 6 Department of Microbiology, Victoria Infirmary, Hayfield Road, Kirkcaldy, KY2 5AH, UK; 7 Infection Unit, University Hospital Monklands, Monkscourt Avenue, Airdrie, ML6 OJS, UK; 8 Antimicrobial Management Team, Aberdeen Royal Infirmary, Foresterhill Health Campus, Foresterhill Road, Aberdeen, AB25 2ZN, UK; 9 Antimicrobial Management Team, University Hospital Crosshouse, Kilmarnock Road, Crosshouse, Kilmarnock, KA2 0BE, UK; 10 Respiratory Medicine Unit, Raigmore Hospital, Old Perth Road, Inverness, IV2 3UJ; 11 Regional Infectious Diseases Unit, Western General Hospital, Crewe Road South, Edinburgh, EH4 2XU, UK; 12 Pharmacy Department, Ninewells Hospital, James Arrott Drive, Dundee, DD2 1SG, UK

## Abstract

**Background:**

Bacterial co-infection is infrequently observed with SARS-CoV-2/COVID-19 infection outside of critical care, however, antibiotics are commonly prescribed.

**Objectives:**

To examine factors associated with antibiotic prescribing for suspected respiratory tract infection (RTI) and evaluate the nature and dynamics of prescribing in hospitalized patients with suspected and proven COVID-19 infection.

**Methods:**

An antibiotic point prevalence survey in hospitalized adult patients was conducted in designated COVID-19 clinical areas (including critical care) in 15 Scottish hospitals. Antibiotics prescribed for RTI and factors associated with prescribing were investigated.

**Results:**

Of 820 surveyed patients, 272 (prevalence 33.3%) received antibiotics for suspected RTI on the survey day and 58.8% were SARS-CoV-2 positive. Antibiotics were empirical in 91.9% and amoxicillin (24.6%), doxycycline (20.5%) and co-amoxiclav (15%) were most frequently prescribed. Oral antibiotics were prescribed in 54.5% and duration was recorded in 76.7% on wards for a median of 5 days. IV to oral switch occurred after a median of 2 days. Prescribing for RTI was independently and positively associated with COPD/chronic lung disease, purulent/bloody sputum, abnormal chest X-ray, and CRP ≥ 100 mg/L. Probable and definite hospital-acquired COVID-19 and diabetes were associated with a lower odds of receiving an antibiotic for RTI.

**Conclusions:**

Antibiotic prescribing for suspected RTI was commonly observed and predominantly empirical in suspected or proven COVID-19. Initiatives to reinforce stewardship principles including clinical review, effective use of microbiological diagnostics and better understanding of the role of biomarkers are central to further limit unnecessary antibiotic therapy in COVID-19.

## Introduction

Lower respiratory tract infection (RTI) is the commonest indication for antibiotics in hospitalized adults in Scotland, accounting for more than one in three antibiotic prescriptions.^1^ Limiting unnecessary antibiotic prescribing in viral or low-risk bacterial RTI is a key focus for antimicrobial stewardship (AMS). The emergence of Severe Acute Respiratory Syndrome Coronavirus 2 (SARS-CoV-2) and the COVID-19 pandemic has significantly challenged AMS efforts as empirical antibiotic prescribing has become widespread.^2^^,^^3^ In March 2020, the Scottish Antimicrobial Prescribing Group (SAPG) developed and disseminated AMS advice via local Antimicrobial Management Teams (AMTs) cautioning against routine antibiotic use in COVID-19 without clinical or radiological evidence of bacterial infection. The advice also promoted the judicious use of short duration (5 days) narrow-spectrum antibiotics such as doxycycline and amoxicillin in patients with clinical suspicion of pneumonia or bacterial lower RTI.^4^ A point prevalence survey (PPS) of antimicrobial use was conducted at the time of peak SARS-CoV-2 clinical activity in patients with suspected or proven COVID-19 infection in Scottish hospitals. We observed a 45.0% prevalence of antibiotic prescribing in this population, with suspected RTI being the commonest recorded indication (in 73.9%).^5^ Herein we explore variables associated with antibiotic prescribing specifically in this subset of the population with suspected RTI and in addition examine key elements of antibiotic prescribing, including AMS measures not described in the previous report.^5^

## Patients and methods

### Study design and inclusion criteria

A PPS of hospitalized adult patients with suspected or confirmed COVID-19 was conducted between 20 and 30 April 2020 in designated COVID-19 clinical areas (wards and critical care units) across 15 NHS Scotland hospitals. PPS methodology has been described in full elsewhere.^5^ The diagnosis of RTI was as recorded in case records and included any of pneumonia (hospital-acquired, community-acquired, aspiration or ventilator-associated), infective exacerbation of COPD or other lower RTI. Data were collected from handwritten and electronic medical and laboratory records and prescription charts.

### Data definitions

Probable nosocomial COVID-19 and definite nosocomial COVID-19 were defined as a positive SARS-CoV-2 test, 8–14 days and more than 14 days following admission, respectively.^6^

Information on antibiotics prescribed for suspected RTI on the day of data collection including start date, route, and whether IV to oral switch had occurred was recorded. Prescribing was recorded as empirical (prior to microbiological confirmation) or directed (following microbiological confirmation). Specific microbiological results were not collected. Potential factors influencing antibiotic prescribing were recorded as were descriptive clinical data, including: presence and nature of sputum production (grouped as ‘purulent/bloody’ if recorded as green, brown or bloody sputum); C-reactive protein (CRP) value; and chest X-ray results were grouped as normal or abnormal (including COVID-19 compatible, indeterminate, pneumonia or other abnormal). Other non-infection clinical management and advanced care planning were recorded where available. Anonymized data were sent to the core survey team for curation, validation and analysis.

### Descriptive analysis

Analyses included patients with a SARS-CoV-2 Reverse Transcriptase-Polymerase Chain Reaction (RT-PCR) test result. Prevalence estimates with 95% confidence intervals (95% CI) were calculated and frequency tables of survey population and prescribing characteristics were produced. Patients with missing data were excluded from denominators. Data were examined for the whole patient population and comparisons were made between those prescribed antibiotics for RTI and those who did not receive an antibiotic on the day of data collection. Those on medical or elderly care wards (combined as ‘wards’) were compared with those on high dependency and ICUs (combined as ‘critical care’). Pearson’s Chi square tests with a continuity correction or Fisher’s Exact tests were used to compare percentages between two groups and determine if significantly different. A Mann–Whitney U test was used to compare median ages between groups. Median durations were presented with range (minimum to maximum) and interquartile range (IQR). Statistical significance was set at *P < *0.05. All analyses were carried out using R (version 3.5.1).

### Statistical analysis

Univariable and multivariable regression analyses were conducted to identify factors associated with prescribing of antibiotics for RTI on the day of data collection. A survey-weighted binomial model was used (which accounted for clustering of beds within wards).

Univariable factors were screened and those with *P* values below 0.3 were included in a backward elimination and forward stepwise approach to select the most parsimonious multivariable model. Statistical significance was set at *P < *0.05. A category-level *P* value (using the Wald test), odds ratios (OR) and 95% CI were calculated for each factor in the final model.

### Ethics approval

Local governance processes for audit/survey of clinical practice were followed. No patient identifiers were collected by the central survey team and there were no interventions or patient contact during the survey.

## Results

Of 820 patients surveyed,^5^ 272 received at least one antibiotic for suspected RTI on the survey day (prevalence 33.3%, 95% CI = 30.1–36.6). The majority of treated RTI patients (69.5%) were managed in a medical ward, 13.2% in elderly care and 17.3% in critical care. On the day of data collection 160 (58.8%) were SARS-CoV-2 positive. Clinical and demographic characteristics of those receiving antibiotics for suspected RTI are shown in Table 1. A total of 347 antibiotics were prescribed in 272 patients. Most frequent were amoxicillin (24.6%), doxycycline (20.5%), amoxicillin/clavulanate (15%) and piperacillin/tazobactam (6.9%). Macrolides were prescribed in 6.8% and fluoroquinolones and cephalosporins were prescribed 3.4% and 0.3%, respectively. Meropenem and piperacillin/tazobactam comprised 21.7% and 18.3% of antibiotics prescribed in critical care units and 1.0% and 4.5% of those in wards, respectively (Table 2). Single agent therapy was used in 74.9%, whilst 22.5% received dual, and 2.6% received triple therapy (Table 3). Antibiotic prescribing for RTI on the day of data collection was empirical in 91.9% of patients (Table 3). Overall, 146 (54.5%) patients with suspected RTI were receiving oral antibiotic therapy (64.1% on wards and 6.7% in critical care). Planned duration of oral antibiotic therapy was recorded in 76.7% of patients on wards and median recorded planned duration of oral antibiotic therapy was 5 days (range 2–10, IQR 3–7 days). Patients who had switched from IV to oral did so after a median IV duration of 2 days (range <1–18, IQR 1–4 days). Of those receiving IV therapy, the median duration of IV antibiotics was 2 days (range 1–26, IQR 2–3) on wards and 3 days (range 1–13, IQR 2–6) on critical care units.

**Table 1.** Characteristics of the population prescribed antibiotics for respiratory tract infection (RTI)

	Patients receiving antibiotic for RTI on day of survey (*n = *272)		
Patient characteristics	Total surveyed patients	Total surveyed with characteristic	Percentage
COVID status			
SARS-CoV-2 positive test	272	160	58.8
Probable or definite nosocomial COVID-19	272	16	5.9
COVID-19 suspected on admission	272	247	90.8
Demographics			
Age, median (range)	68.5 years (range 17–95, IQR 56–79)		
Sex (% male)	272	138	50.7
Location			
Care home resident	272	25	9.2
Ward type—Critical care	272	47	17.3
Ward type—Elderly	272	36	13.2
Ward type—Medical	272	189	69.5
Comorbidities			
Asthma	272	35	12.9
Cardiovascular disease	272	87	32.0
COPD/chronic lung disease	271	76	28.0
Diabetes	272	49	18.0
Hypertension	271	106	39.1
Immunocompromised	272	37	13.6
Long term renal dialysis	272	5	1.8
Morbid obesity	267	20	7.5
Other chronic condition	270	165	61.1
Other			
Other suspected infection	271	140	51.7
Penicillin allergy	272	42	15.4
Diagnostics/clinical signs			
Abnormal chest X-ray	272	224	82.4
CRP ≥ 100 mg/L	269	130	48.3
Purulent/bloody sputum	272	40	14.7
Management			
Clinical therapeutic trial	272	39	14.3
Supplemental oxygen	272	158	58.1
Treatment escalation^a^	229	110	48.0
DNAR recorded	271	132	48.7

COPD, chronic obstructive pulmonary disease and other chronic lung disease; DNAR, do not attempt cardiopulmonary resuscitation order; CRP, C-reactive protein.

a Treatment escalation: recorded treatment plan including planned multi-disciplinary team discussion.

**Table 2.** Antibiotics prescribed for suspected respiratory tract infection, by ward type and by SARS-CoV-2 result

Antibiotics prescribed on day of survey	Total number of prescriptions	Percentage of total	Medical and elderly wards		Critical care	
			SARS-CoV-2 positive	SARS-CoV-2 negative	SARS-CoV-2 positive	SARSCoV-2 negative
Amoxicillin	85	24.6	35	45	5	0
Azithromycin	5	1.4	1	2	2	0
Aztreonam	1	0.3	0	0	1	0
Ceftriaxone	1	0.3	1	0	0	0
Ciprofloxacin	5	1.4	1	2	2	0
Clarithromycin	19	5.5	5	10	3	1
Amoxicillin/clavulanate	52	15.0	22	21	8	1
Trimethoprim/ sulfamethoxazole	9	2.6	6	3	0	0
Doxycycline	71	20.5	41	30	0	0
Flucloxacillin	2	0.6	1	0	1	0
Gentamicin	11	3.2	8	3	0	0
Levofloxacin	7	2.0	4	2	1	0
Meropenem	16	4.6	2	1	12	1
Metronidazole	16	4.6	5	10	0	1
Other	7	2.0	0	3	2	2
Piperacillin/tazobactam	24	6.9	7	6	10	1
Temocillin	4	1.2	1	1	2	0
Trimethoprim	0	0.0	0	0	0	0
Vancomycin	11	3.2	5	2	4	0
Total	346^a^	100.0	145	141	53	7

a Patients may have received more than one antibiotic. The name of one antimicrobial was not recorded for one patient (COVID-19 positive and in critical care) receiving an antibiotic on the day of the survey for a respiratory indication.

**Table 3.** Antibiotic use in patients with respiratory tract infection (RTI) on day of survey

	Medical and elderly wards (*n = *697)			Critical care wards (*n = *121)			Total (*n = *818)		
Characteristic	Total surveyed patients	Total surveyed patients with characteristic	%	Total surveyed patients	Total surveyed patients with characteristic	%	Total surveyed patients	Total surveyed patients with characteristic	%
Antibiotic(s) for RTI	697	225	32.3	121	47	38.8	818	272	33.3
Antibiotic(s) for RTI (% all treated)	314	225	71.7	54	47	87.0	368	272	73.9
Empirical antibiotic for RTI	224	213	95.1	47	36	76.6	271	249	91.9
SARSCoV-2 positive and RTI antibiotic(s)	225	117	52.0	47	43	91.5	272	160	58.8
Single or multiple antibiotic(s) for RTI									
Single antibiotic	225	170	75.6	46	33	71.7	271	203	74.9
Dual antibiotics	225	49	21.8	46	12	26.1	271	61	22.5
Triple antibiotics	225	6	2.7	46	1	2.2	271	7	2.6
Total antibiotics prescribed (all indications)	420			70			490		
Total antibiotics prescribed for RTI	286			61			347		
Route of administration of all antibiotics									
Oral	283	174	61.5	59	6	10.2	342	180	52.6
IV	283	109	38.5	59	53	89.8	342	162	47.4
No. receiving IV antibiotic(s)	223	80	35.9	45	42	93.3	268	122	45.5
No. Treated with oral therapy only	223	143	64.1	45	3	6.7	268	146	54.5
No. receiving oral following IV antibiotic	135	48	35.6	2	1	50	146	49	33.6
Median duration of IV antibiotic (at time of survey)	2 days (range 1–26, IQR 2–3)			3 days (range 1–13, IQR 2–6)			2 days (range 1–26, IQR 2–4)		
Median duration of IV antibiotic prior to oral for RTI antibiotics	2 days (range <1–14, IQR 1–3)			8 day (range 2–18, IQR 5–12)			2 days (range <1–18, IQR 1–4)		
No. patients where proposed duration of oral antibiotic was recorded for RTI	112/146 (76.7%)			NA			112/146 (76.7%)		
Median recorded planned duration of oral antibiotic for RTI	5 days (range 2–10, IQR 3–7)			NA			5 days (range 2–10, IQR 3–7)		

NA, not applicable.

Clinical variables were examined for an association with antibiotic prescribing for RTI (versus no antibiotic prescription) on the day of data collection (Table 4). In a multivariable logistic regression analysis, prescribing for RTIs was independently and positively associated with COPD/chronic lung disease, purulent/bloody sputum, an abnormal chest X-ray, and CRP ≥ 100 mg/L. SARS-CoV-2 RT-PCR-positive, probable and definite hospital-acquired COVID-19 and diabetes were all associated with lower odds of receiving an antibiotic for RTI on the day of survey (Table 5).

**Table 4.** Univariate logistic regression analysis of factors associated with either antibiotic prescribing for respiratory tract or no antibiotic prescribing on the day of survey

Risk factor theme/risk factor	Category^a^	Odds ratio (OR)	OR 95% Lower CI	OR 95% Upper CI	Category *P* value	Risk factor *P* value
COVID-19 status						
SARS-CoV-2 test result	Negative*	1.00				
	Positive	0.50	0.32	0.78	0.003	0.003
Probable/definite nosocomial COVID-19 (>day 8)	No*	1.00				
	Yes	0.24	0.14	0.43	<0.001	<0.001
Positive SARS-CoV-2 test result prior to admission	No*	1.00				
	Yes	1.40	0.68	2.89	0.36	0.36
Positive SARS-CoV-2 test from admission to day 7	No*	1.00				
	Yes	0.86	0.61	1.21	0.40	0.40
Demographics						
Age	Cont.	0.99	0.98	1.00	0.01	0.01
Sex	Female*	1.00				
	Male	0.92	0.66	1.29	0.62	0.62
Location						
Care home resident	No*	1.00				
	Yes	1.03	0.63	1.69	0.89	0.89
Ward type	Critical care*	1.00				
	Elderly	0.42	0.21	0.84	0.02	
	Medical	0.94	0.60	1.45	0.77	0.04
Length of stay from admission to survey						
Length of stay	Cont.	0.97	0.96	0.99	0.008	0.008
Comorbidities						
Asthma	No*	1.00				
	Yes	1.65	0.99	2.74	0.06	0.06
Cardiovascular disease	No*	1.00				
	Yes	1.00	0.70	1.40	0.96	0.96
COPD/Chronic lung disease	No*	1.00				
	Yes	2.59	1.86	3.60	<0.001	<0.001
Diabetes	No*	1.00				
	Yes	0.56	0.39	0.79	0.001	0.001
Hypertension	No*	1.00				
	Yes	0.96	0.63	1.46	0.85	0.85
Immunocompromised	No*	1.00				
	Yes	1.22	0.74	1.99	0.44	0.44
Long term renal dialysis	No*	1.00				
	Yes	0.82	0.30	2.27	0.71	0.71
Morbid obesity	No*	1.00				
	Yes	1.00	0.57	1.76	1.00	1.00
Other chronic illness	No*	1.00				
	Yes	0.98	0.73	1.32	0.90	0.90
Treatment escalation						
DNAR	No*	1.00				
	Yes	0.70	0.49	1.02	0.07	0.07
Diagnostics/clinical signs						
Chest x-ray	Normal*	1.00				
	Abnormal	2.14	1.49	3.08	<0.001	<0.001
CRP	0–99 mg/L	1.00				
	≥100 mg/L	1.79	1.32	2.41	<0.001	<0.001
Sputum	Normal*	1.00				
	Purulent or bloody	2.33	1.54	3.54	<0.001	<0.001

COPD, chronic obstructive pulmonary disease and other chronic lung disease; DNAR, do not attempt cardiopulmonary resuscitation order; CRP, C-reactive protein. Modelling excludes records with unknown antimicrobial status, COPD/chronic lung disease, morbid obesity, treatment for high blood pressure, cardiovascular disease, immunocompromised as per HPS/SG advice, other chronic illness and DNAR leaving 694 rows for inclusion in the analysis.

a An asterisk denotes the reference category.

**Table 5.** Multivariable analysis of factors associated with antibiotic prescribing for a respiratory tract infection indication^a^^,^^b^

Variable	OR	Lower 95% CI	Upper 95% CI	Wald test *P* value
SARS-CoV-2 positive	0.51	0.33	0.81	0.005
COPD/chronic lung disease	2.40	1.66	3.46	<0.001
Diabetes	0.58	0.40	0.84	0.006
CRP ≥ 100 mg/L	1.83	1.28	2.61	0.001
Abnormal chest X-ray	1.88	1.22	2.90	0.005
Purulent or bloody sputum	1.85	1.17	2.91	0.01
Probable or definite nosocomial COVID-19	0.43	0.24	0.74	0.004

a Patients who were prescribed antibiotics for other indications were excluded from this analysis.

b Modelling excludes records with unknown antimicrobial status, COPD/chronic lung disease, morbid obesity, treatment for high blood pressure, cardiovascular disease, immunocompromised as per HPS/SG advice, other chronic illness and DNAR, leaving 694 rows for inclusion in the analysis.

## Discussion

We previously observed that suspected RTI was the commonest indication for antibiotics in hospitalized adult patients with suspected or proven COVID-19.^5^ In this analysis we have examined the detail of antibiotic prescribing within the RTI group and explored factors associated with receipt of an antibiotic prescription. We observed that antibiotics recommended in local guidelines for lower RTI (amoxicillin, doxycycline and amoxicillin/clavulanate) accounted for three of every five antibiotic prescriptions for RTI. Broader-spectrum agents (piperacillin/tazobactam and meropenem) were largely reserved for those in critical care units, reflecting suspected nosocomial/ventilation-associated infection.^5^ SAPG COVID-19 antibiotic guidance promotes oral antibiotics over IV therapy when clinically appropriate and IV to oral switch when clinical improvement when initial IV therapy is used.^4^ Approximately two-thirds of those receiving antibiotics for RTI on wards were receiving oral therapy and one-third of those had switched from IV therapy after a median of 2 days suggesting timely review and rationalization of therapy. Of those prescribed IV therapy on wards on the day of the data collection, the median IV therapy duration was 2 days suggesting prolonged IV antibiotic therapy in ward-based patients was exceptional and indicates adherence to IV to oral switch guidance. Short duration therapy (5 days) for lower RTI was promoted prior to the COVID-19 pandemic and was emphasized again to Antimicrobial Management Teams by SAPG in March 2020^4^ in the context of the pandemic.

It is recommended to record the duration of oral antibiotic therapy on the drug prescription chart/electronic record and we observed this in the majority (77.0%) of cases. Recorded planned duration of oral therapy also aligned with national and local guidance, with a median duration of 5 days.

Variables associated with an antibiotic prescription for presumptive lower RTI likely reflects clinical uncertainty and inability to rule out bacterial infection.^5^ Those with proven SARS-CoV-2 and probable or definite nosocomial COVID-19 were less likely to be receiving an antibiotic, likely reflecting less clinical uncertainty, rationalization of treatment following review of test results. No differences in antibiotic prescribing for RTI were observed between those in whom decisions were documented for ‘ward level care’ or ‘not for resuscitation’ supporting the operational policy that ‘not for resuscitation’ does not mean ‘not for any treatment’. The importance of antibiotic decision making at the end of life has been the focus of both a recent systematic review^7^ and the SAPG Good Practice Recommendations,^8^ which emphasize the importance of agreed treatment goals, shared decision making and regular antibiotic review. Further work to better understand therapeutic decision making at end of life in COVID-19 would be useful.

COVID-19 continues to challenge antimicrobial stewardship efforts, with a lack of published evidence to support a role for bacterial coinfection during the early (pre-critical care) phase of SARS-CoV-2.^9–11^ We found CRP to be an independent predictor for an antibiotic prescription for RTI in suspected and proven COVID-19. In contrast to most other respiratory viral infections CRP lacks utility in differentiating bacterial from SARS-CoV-2 infection and its use during the pandemic reflects prescribing practice pre-pandemic. The biomarker procalcitonin (PCT) is not routinely measured outside of critical care in NHS Scotland. PCT is often elevated in the inflammatory response to SARS-CoV-2 as well as in the context of bacterial RTI.^12^ Although PCT cannot currently be recommended as a guide to the initiation of antibiotics in the context of COVID-19, there may be value in using it to guide early discontinuation of antibiotics,^13^ particularly in critical care patients when used in combination with careful microbiological assessment. Knowledge of the low prevalence of bacterial co-infection, together with rapid SARS-CoV-2 diagnosis including point of care testing, should reduce clinical uncertainty in antibiotic decision-making and the use of corticosteroids in patients requiring supplemental oxygen therapy offers an effective therapeutic advance for hospitalized patients.^14^ Unfortunately, a proportion of high-risk patients with COVID-19 will deteriorate and nosocomial infection, particularly ventilator-associated pneumonia, will continue to be a significant challenge.^15^ Judicious use of antibiotics in ward patients and careful microbiology isolate-driven prescribing in the critical care setting should limit unnecessary escalation of antimicrobials and limit the future risk of antimicrobial-resistant and opportunistic infections.

Although we have demonstrated evidence supporting good antibiotic stewardship in the context of suspected RTI in COVID-19, antibiotic use prevalence in the absence of evidence of bacterial co-infection remains a concern. Our survey was also limited as it did not include microbiological data, but data collectors did record whether prescribing was microbiologically directed or empirical. The rates of observed empirical antibiotic prescribing for RTI highlights the importance of ongoing local and national initiatives to reinforce stewardship in the COVID-19 context and beyond. Clinical review and decision making, and early use of microbiological diagnostics, are central to limit unnecessary antibiotic therapy and other antibiotic-related harm.
